# 2nd interventions in aging conference

**DOI:** 10.18632/aging.101221

**Published:** 2017-04-27

**Authors:** Brian K. Kennedy, Linda Partridge

**Affiliations:** ^1^ Buck Institute for Research on Aging, Novato, CA 94945; ^2^ Departments of Biochemistry and Physiology, Yong Loo Lin School of Medicine, National University Singapore, 117597, Singapore; ^3^ Institute for Healthy Ageing and Department of Genetics, Evolution and Environment, University College London, London, WC1E 6BT UK; ^4^ Max Planck Institute for the Biology of Ageing, Köln, 50931, Germany

**Keywords:** healthspan, organismal aging, epigenetics, longevity, cellular mechanisms, metabolism

In March 2017, the Second Interventions in Aging Conference was held in Cancun, Mexico and organized by Fusion Conferences, Ltd. As meeting organizers, we would like to provide a brief overview of the meeting proceedings, which reflect larger views in the aging research field about the future directions critical for continued progress.

The meeting, similar to the earlier event in 2015, was focused on interventional strategies. One notable difference was that this year's meeting was much more directed toward potential interventions to target human aging. The field has been very successful over the last decade in identifying interventions that extend lifespan and healthspan in animal models such as yeast, flies, worms, mice and, to some extent, primates. However, the primary goal is to employ knowledge from basic aging research to develop novel medical strategies aimed at extending human healthspan. Aging is the biggest risk factor for a wide range of chronic diseases that, to date, medical strategies have treated as separate entities, and as they arise. Yet, aging is driven by a limited number of coordinated pathways that can be modulated, and evidence suggests that interventions delaying aging will protect against multiple age-related diseases simultaneously [1,2]. Discoveries in basic aging research thus point towards a broad-spectrum, preventative, medical strategy for aging-related disease.

There were seven research topics each addressed thematically at the meeting. All were chosen because they embody different strategies to target human aging. Each session combined talks from Platform speakers with those chosen from submitted abstracts. The first and largest theme was targeted toward *Organismal Aging*, or understanding the intrinsic pathways that govern aging of the entire organism. Platform presentations were provided by Rozalyn Anderson (University of Wisconsin-Madison), Adam Antebi (Max Planck Institute, Köln), Holly Brown-Borg (University of North Dakota), Yuki Ikeno and Nicolas Musi (University of Texas Health Sciences Center), Laura Niedernhofer (Scripps Florida), Scott Pletcher (University of Michigan), Eline Slagboom (Leiden University Medical Center). The interesting aspect of these presentations is that they address strategies to modify aging that touch back to research from the early days of aging research while simultaneously pointing to novel strategies for future interventions. Dr. Brown-Borg defined new mechanisms linking growth hormone signaling to aging, Dr. Ikeno, using mammalian models, re-evaluated the role of reactive oxygen species; Dr. Niedernhofer presented new evidence for links between progeria and normal aging, interpreting these strategies in the context of possible interventions that may affect both normal and “premature” aging; Dr. Musi linked NFKB signaling to sarcopenia, a major driver of frailty in aging; Dr. Pletcher described fly studies to examine the impacts of psychological stress on aging using flies; Dr. Antebi discussed novel strategies linking nuclear structure to aging using the classic model organism – *C. elegans*; Dr. Anderson elaborated mechanisms linking calorie restriction to lifespan extension in primates; and Dr. Slagboom described strategies to examine the impact of aging pathways in elderly human populations.

The second theme was focused on using *Stem Cells* to target aging, with exciting presentations by Heinrich Jasper (Buck Institute for Research on Aging) on aging of epithelial stem cells in flies and mice, Emmanuelle Passague (Columbia University) on links between metabolism, autophagy and aging in the hematopoietic system and Sara Wickstrom (Max Planck Institute, Köln), who discussed focused on how adult stem cells self-organize into functional configurations. The third theme, addressing *Cellular Mechanisms of Longevity Assurance*, focused on pathways suspected to modulate aging, including autophagy by Malene Hansen (Sanford Burnham Prebys Medical Discovery Institute), mitochondrial function and aging with emphasis on the role of small mitochondrial peptides by David Lee (University of Southern California), and the hypoxia pathway by Dana Miller (University of Washington).

Theme 4 centered on *Epigenetics*, which is not only becoming a target for intervention in aging, but is rapidly becoming a leading candidate for providing biomarkers of biological age. Weiwei Dang (Baylor College of Medicine) studied mesenchymal stems cells and adipocyte differentiation, elucidating mechanisms leading to activation of the protein deacetylase, SIRT1; Dr. Eric Greer (Harvard Medical School/Boston Children's Hospital) evaluated mechanisms leading to transgenerational inheritance of epigenetic marks that impact lifespan, John Sedivy (Brown University) described links between the epigenome and activation of somatic retrotransposons, and how this activation may drive senescence and aging; and Steve Horvath (UCLA) detailed a number of studies further promoting the epigenetic clock as a marker of accelerated and delayed aging.

Theme 5 was designed to take a *Systems Aging* viewpoint. Such a holistic understanding of the aging process is in a sense the ultimate goal of the research. Is it possible to understand such a complex process as aging not just one gene and pathway at a time but in totality? Anne Brunet (Stanford University) discussed strategies to model aging using worms and killifish (a short-live vertebrate gaining popularity in the aging research field); Paul Robbins (Sripps Florida) described studies to target mammalian aging and Christiaan Leeuwenburgh (University of Florida) described the multi-fold connections between iron metabolism/transport and aging. The final theme centered on *Signaling and Metabolism*, hitting the major metabolic pathways that are linked to aging and that can be targeted with interventional strategies. These include the mTOR pathway and rapalogs, discussed by Matt Kaeberlein (University of Washington); dietary restriction and links through mTOR to regulation of mRNA splicing, discussed by William Mair (Harvard University), dietary restriction in primates discussed by Julie Mattison (National Institute on Aging/NIH), NAD metabolism and Sirtuins, discussed by David Sinclair (Harvard Medical School) and mitochondrial roles in regulating aging and metabolism, discussed by Pinchas Cohen (USC Leonard Davis School of Gerontology).

As we move closer to interventions targeting human aging, it is critical to get together and discuss the best strategies and the ideal path forward. The meeting in Cancun provided a low stress atmosphere where scientists in the aging field could not only share data but take the time to think creatively and discuss issues in depth. We as organizers thank the session chairs, speakers, poster presenters and attendees, as well as the staff of Fusion Conferences, Ltd. for enabling a fascinating meeting. We also look forward to the 3^rd^ Interventions in Conference to be held in approximately two years.

The Second Interventions in Aging Conference was organized by Fusion Conferences, Ltd.

**Figure:**
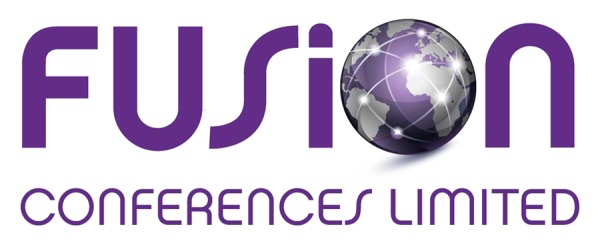


The Second Interventions in Aging Conference was sponsored by Aging (www.aging-us.com).

Aging is published by Impact Journals, LLC.

**Figure:**
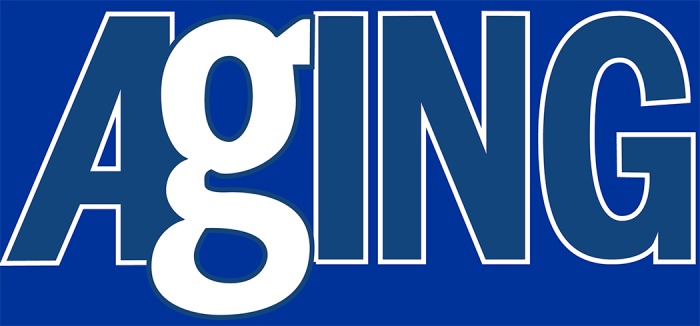


## CONFERENCE PHOTOS

**Figure:**
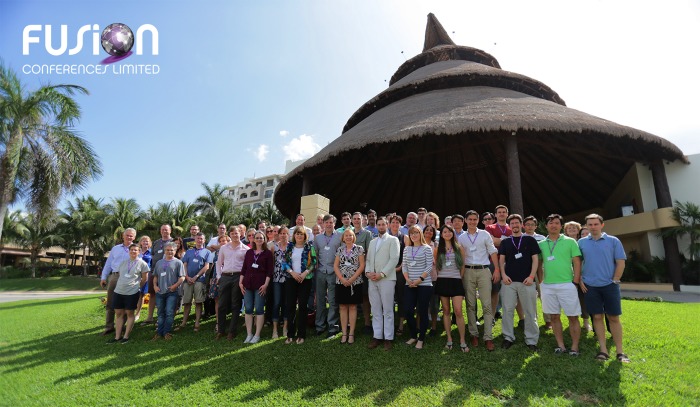
Group photo of Conference participants

**Figure:**
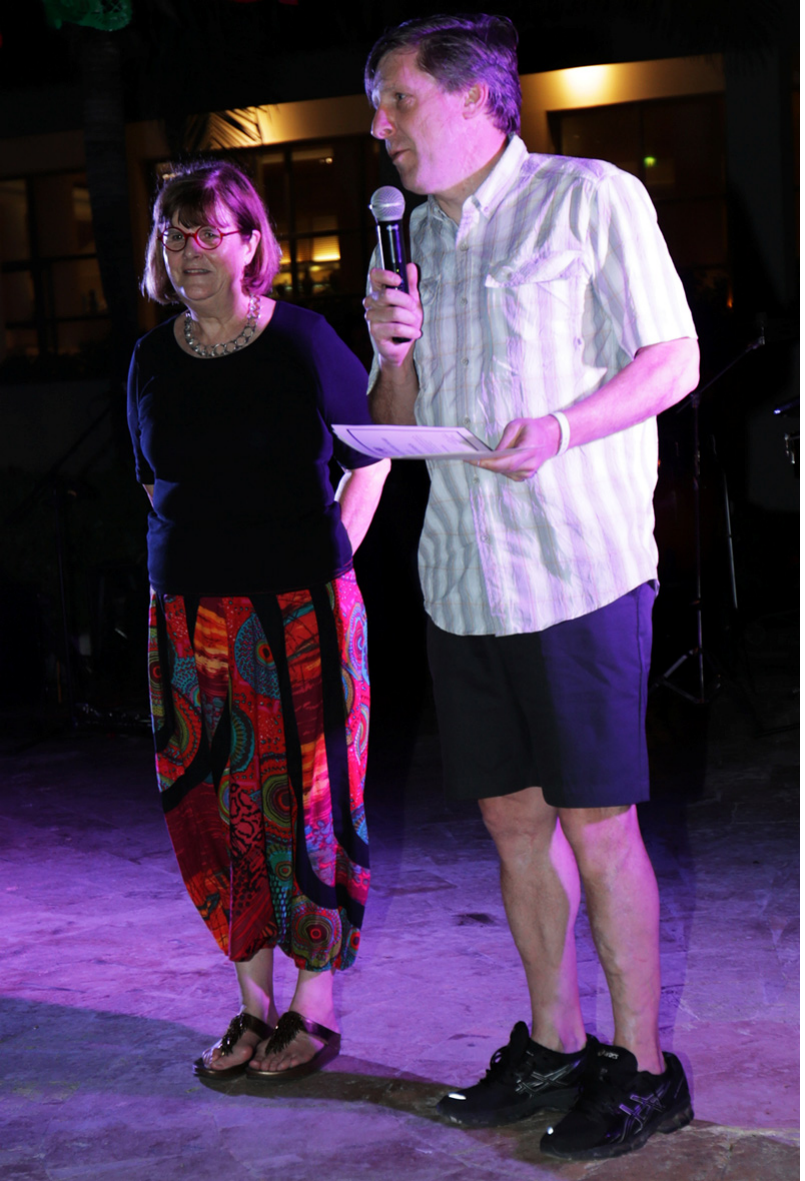
Linda Partridge and Brian Kennedy

**Figure:**
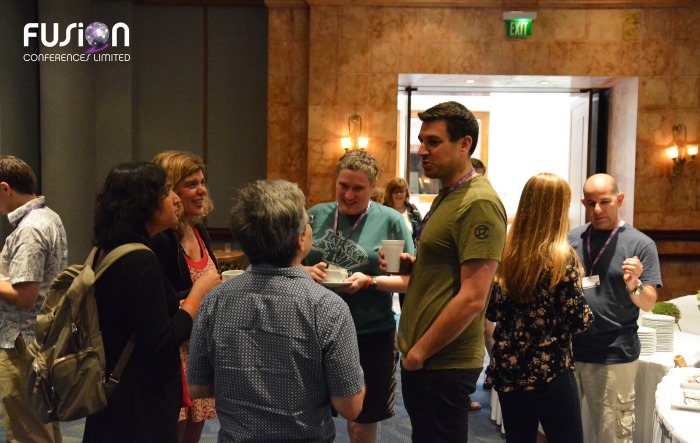
Scientific discussion

**Figure:**
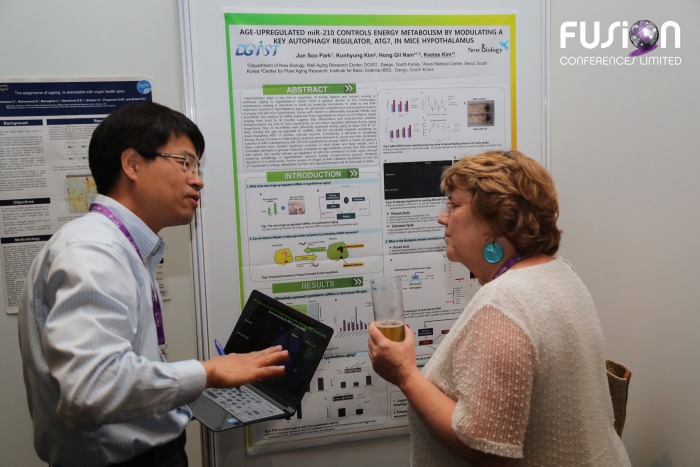
Keetae Kim and Eline Slagboom

**Figure:**
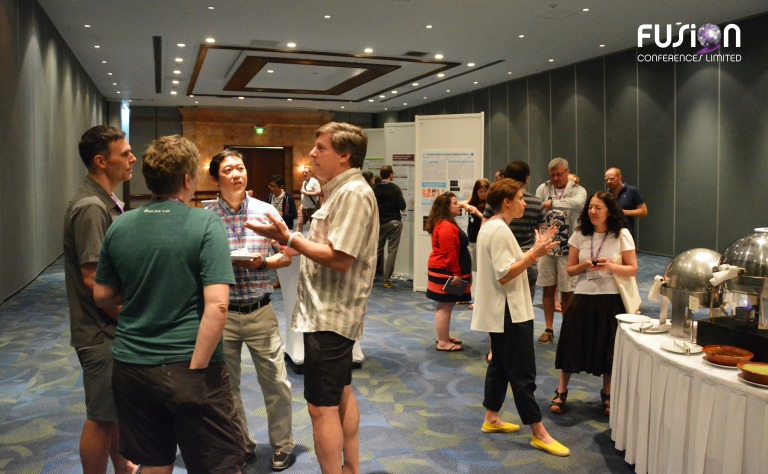
Poster session

**Figure:**
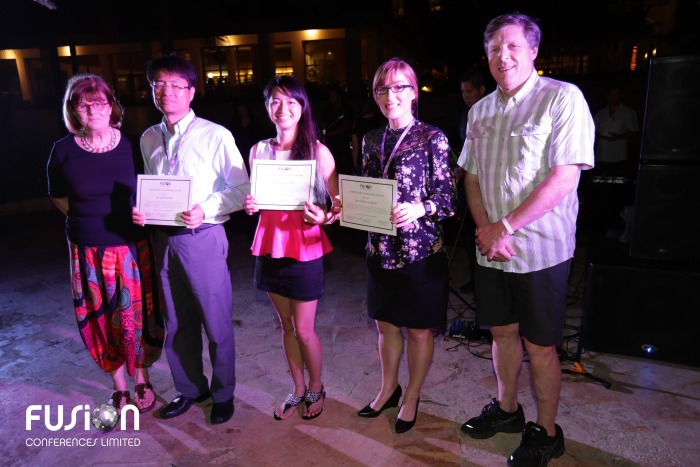
Poster competition winners

**Figure:**
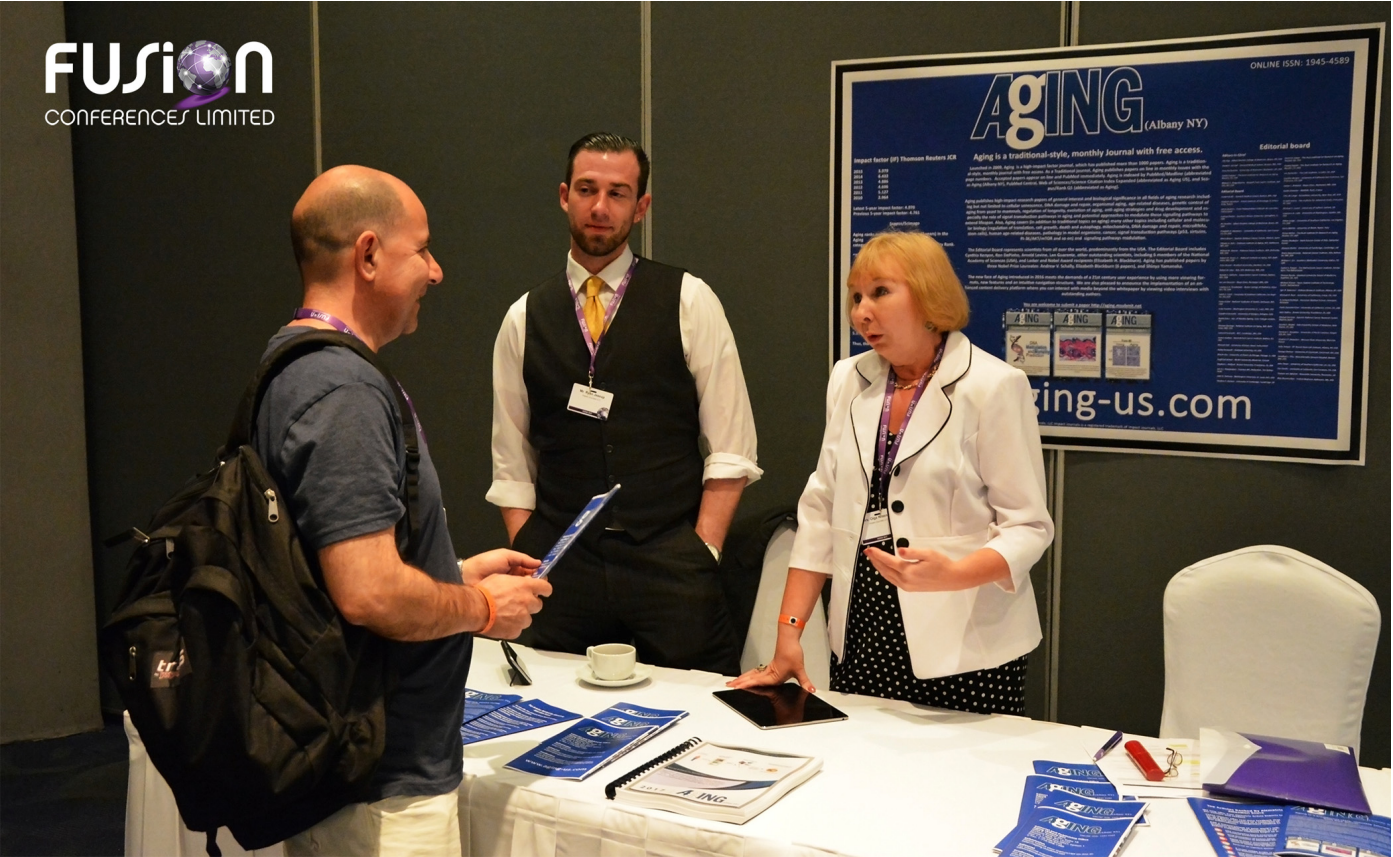
Aging Conference sponsors
